# Giant Prolactinoma of Young Onset: A Clue to Diagnosis of MEN-1 Syndrome

**DOI:** 10.1155/2018/2875074

**Published:** 2018-08-14

**Authors:** Chandrika Jayakanthi Subasinghe, Noel Somasundaram, Pathmanathan Sivatharshya, Lalana Devi Ranasinghe, Márta Korbonits

**Affiliations:** ^1^Endocrinology Unit, National Hospital of Sri Lanka, Colombo, Sri Lanka; ^2^Department of Endocrinology, William Harvey Research Institute, Barts and the London School of Medicine and Dentistry, Queen Mary University of London, UK

## Abstract

Multiple endocrine neoplasia (MEN) type 1 syndrome is an autosomal dominant disorder caused by germline mutations in* MEN1* gene, characterized by tumours in endocrine and nonendocrine organs. Giant prolactinoma is defined as tumours larger than 40mm with very high prolactin secretion. We report two unrelated Sri Lankan patients (8-year-old boy and a 20-year-old female) who presented with giant prolactinomas with mass effects of the tumours. The female patient showed complete response to medical therapy, while the boy developed recurrent resistant prolactinoma needing surgery and radiotherapy. During follow-up, both developed pancreatic neuroendocrine tumours. Genetic analysis revealed that one was heterozygous for a nonsense mutation and other for missense mutation in* MEN1* gene. Screening confirmed familial MEN-1 syndrome in their families. High clinical suspicion upon unusual clinical presentation prompted genetic evaluation in these patients and detection of* MEN1* gene mutation. Pituitary adenomas in children with MEN-1 syndrome are larger tumours with higher rates of treatment resistance. This report emphasizes importance of screening young patients with giant prolactinoma for MEN-1 syndrome and arranging long-term follow-up for them expecting variable treatment outcomes. Sri Lanka requires further studies to describe the genotypic-phenotypic variability of MEN-1 syndrome in this population.

## 1. Background

Giant prolactinoma is defined as a pituitary tumour with a largest diameter of 40 mm or more in any direction with massive extrasellar extension and very high prolactin levels (usually above 1000 *μ*g/L) and no concomitant GH or ACTH secretion [1–4]. These are rare, accounting for only 2-3% of prolactinomas with a male to female ratio of 9:1. Prevalence of giant prolactinomas is highest in the fourth to fifth decades, and they often respond to dopamine agonist (DA) therapy [4]. According to a recently published literature review, giant prolactinomas are rare among children and adolescents. In a 2014 review a total of 16 giant prolactinoma cases in children younger than 15 years were found; 15 were boys (age range 6-14 years) with one girl aged 14.5 years [1]. Several authors have recommended genetic testing for aryl hydrocarbon receptor interacting protein (*AIP*) mutation and multiple endocrine neoplasia 1 (*MEN1*) mutation, for isolated sporadic growth hormone (GH) and prolactin (PRL) secreting pituitary tumours in all patients less than 18 years and in patients less than 30 years old with macroadenomas [5, 6]. In a French study, 8.6% of paediatric isolated pituitary macroadenomas were positive for* AIP* mutation, while 3.4% were positive for* MEN1* mutation [6].

MEN-1 syndrome [MEN1; MIM #131100] is an autosomal dominant syndrome characterized by tumours in endocrine and nonendocrine glands with 95% penetrance [7]. According to recently published data from the Dutch MEN-1 study group, 38.1% of MEN-1 syndrome patients harboured a pituitary tumour overall, and in 11% it was the initial presentation [8]. A young (under 21 years) MEN-1 cohort of 160 patients also showed almost similar prevalence (34%) of pituitary tumours [9]. Pituitary tumours in MEN-1 syndrome are usually diagnosed at an earlier age and occur more frequently in females. These are mostly macroadenomas and have a higher degree of aggressiveness and invasiveness according to most of the published data [7, 9–12]. The vast majority of MEN-1 syndrome patients harbour a heterozygous germline mutation in the* MEN1* gene, but a few cases have been identified with a* CDKN1B* mutation and a few have no recognized genetic background. A recent* MEN1* gene mutation update reviewed over 1100 germline and 200 somatic mutations. Among known* MEN1* mutations, 41% are frameshift insertions and deletions, 23% are nonsense mutations, 20% are missense mutations, 9% are splice site defects, and 1% are whole or partial gene gross deletions [13].

Sri Lanka currently lacks facilities for genetic analysis of MEN-1 syndrome, and data on prevalence, phenotype, and genotype variability of MEN-1 syndrome in the country is not known. We report the first two genetically confirmed MEN-1 syndrome cases from Sri Lanka. Both of them presented as isolated sporadic cases of giant prolactinomas at very young age and later are found to have MEN-1 syndrome.

## 2. Case Presentation

### 2.1. Case 1

**Figure 1:**
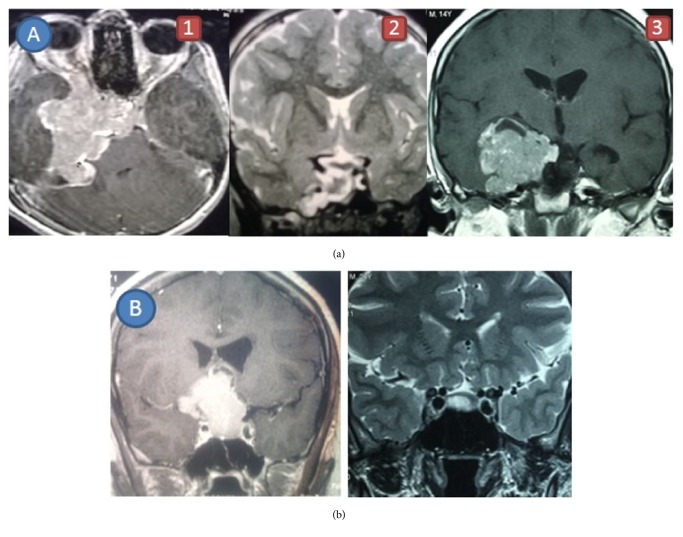
(a) MRI images of Case 1 at diagnosis (1), initial response to dopamine agonist (2), and recurrence of dopamine agonist resistant tumour (3); (b) MRI images of Case2 at diagnosis and showing tumour shrinkage to subcentimeter level after 5 years of dopamine agonist therapy.

An eight-year-old boy initially presented to us in 2008 with progressive headache and visual disturbances. His imaging revealed a giant pituitary tumour (59 x 45 x 42 mm) with extrasellar extension (Figure 1(a)) with initial prolactin of 91,800 *μ*g/L confirming the diagnosis of giant prolactinoma. Initially, he responded well to high doses of cabergoline (7 mg/week) with normalization of prolactin and total tumour shrinkage. A few years later, he developed recurrence of the tumour, which was resistant to cabergoline therapy (Figure 1(a)), and underwent transcranial excision of the tumour in 2013. During the immediate postoperative period, he developed recurrent hypoglycaemic episodes, which was confirmed to be endogenous insulin dependent hypoglycaemia biochemically (insulin was 15.9 *µ*IU/mL and C-peptide was 3.94 ng/mL when random blood glucose was less than 2.1 mmol/L). Imaging located a well circumscribed lesion (20 x 12 x 10 mm) in the head of pancreas. He underwent enucleation of the tumour, and that was confirmed as an insulinoma histologically with benign characteristics (Ki67<1%). Six months after the pituitary surgery he received three-field radiotherapy (4500 cGy) and continued on cabergoline (3.5 mg/week) resulting in declining prolactin levels. His baseline echocardiography was normal. He had normal calcium at presentation, but currently he is being evaluated for new onset primary hyperparathyroidism (total calcium 2.98 mmol/L [normal range: 2.40-2.55], intact PTH 88.2 pg/L [12-60]).

### 2.2. Case 2

A Sri Lankan female first presented in 2006 at the age of 20 years to the emergency department with mass effects of a sellar lesion. She gave a history of intermittent galactorrhoea and secondary amenorrhoea since age of 16 years. She was diagnosed to have a giant prolactinoma (40 x 45 x 30 mm, Figure 1(b)) with hyperprolactinaemia (serum prolactin 8930 ng/dL). She responded well to medical therapy with cabergoline (3.5 mg/week) with normalization of prolactin over 1 year and tumour shrinkage over 5 years (Figure 1(b)). Due to the giant prolactinoma she was suspected to have MEN-1 syndrome and testing for other manifestations was initiated. Her calcium levels were normal but pancreas imaging showed a lesion in the pancreas with a cystic (54 x 53 x 49 mm) and a solid (22 x 24 x 20 mm) component. Biochemical evaluation revealed normal serum gastrin, 24 hour urinary 5 HIAA and chromogranin A level, and negative 72 hour fasting test suggesting it to be a nonfunctional pancreatic neuroendocrine tumours (PNET). She underwent distal pancreatectomy with lymphadenectomy. Histological evaluation confirmed PNET with Ki67<1%. Ten years after her first presentation, she was detected to have asymptomatic hyperparathyroidism with corrected ionized calcium of 1.42 mmol/L [normal range: 1.0-1.3], urinary Ca/Cr ratio of 0.32, and intact PTH level of 98.9 pg/L [12-60].

### 2.3. Genetic Analysis

**Figure 2:**
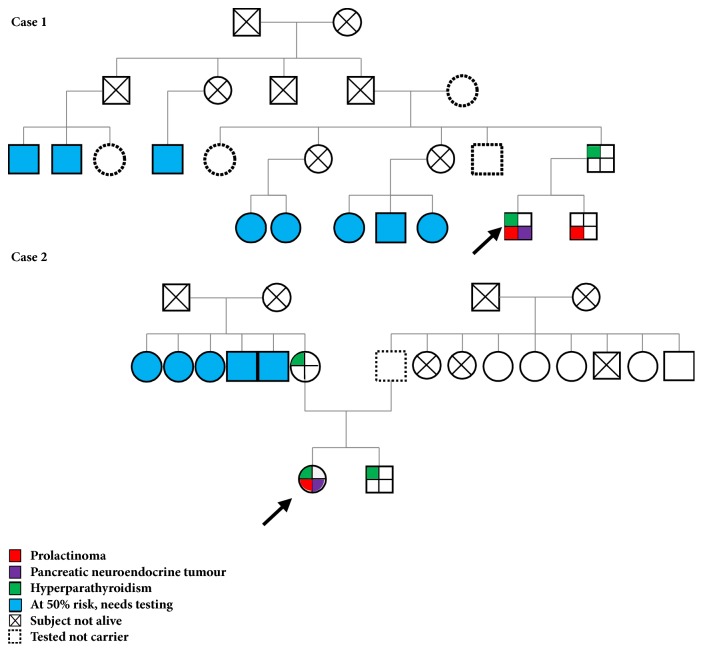
Family trees of the two MEN-1 cases. Cascade genetic testing needs to be followed in both families. Carriers need referral to specialist endocrinology clinic for clinical follow-up. Children of carriers need genetic testing.

Following diagnosis of MEN-1 syndrome with the combination of giant prolactinoma and pancreatic neuroendocrine tumour, both patients underwent genetic analysis for* MEN1 *gene [14] after genetic counselling and obtaining informed written consent. At the time of genetic testing, none of family members from either families showed any evidence of the disease. Case 1 was heterozygous for nonsense mutation in exon 4 of the* MEN1* gene [NM_130799.2, c.781C>T; p.Gln261Ter]; this change has previously been published in a MEN-1 syndrome family [11]. On family screening, the proband's father and brother were positive for the same mutation, and father was found to have hyperparathyroidism (Figure 2). It is unclear at this stage whether the father received the abnormal gene from one of his parents or it was a* de novo* mutation in him. Proband's brother is being followed up for hyperprolactinaemia (38 ng/dL) with normal pituitary imaging.

Case 2 was heterozygous for missense mutation in exon 10 of the* MEN1 *gene [NM_130799.2, c.1736T>C; p.Leu579Pro]; this variant was previously reported in six MEN-1 patients from three Danish families [12]. Same mutation was identified in proband's mother and brother on genetic screening, while her father was found negative. Both the mother and brother had already developed primary hyperparathyroidism of variable severity. Brother had a total calcium of 6.8 mg/dL [normal range: 4.6-5.3], with intact PTH of 240 pg/L [12-60]), and the mother had a corrected ionized calcium of 1.27 mmol/L [normal range: 1.0-1.3] and intact PTH level of 119.4 pg/L). At the time of writing the other hormones are normal and there is no evidence of other endocrine neoplasia in these family members.

Both the families are in the process of undergoing cascade genetic screening (Figure 2). Carriers need endocrine follow-up with screening for clinical, biochemical, and imaging presentation of aspects of the MEN-1 syndrome following available guidelines (Table 1) [7]. Family members with 50% chance to harbour the mutation need genetic testing. Noncarrier family members can be reassured of having chance of develop features of the disease not higher than the general population.

## 3. Discussion

Both of these patients with MEN-1 syndrome presented under the age of 20 years with a giant prolactinoma, in contrast to the usual presentation of such tumours in 4th to 5th decades [1–4]. Early onset of a giant tumour in male patient, in keeping with known literature [1, 2], is thought to be due to rapid growth potential of tumours in males where lower expression of oestrogen receptor alpha may play a role [15]. Young onset giant prolactinomas prompted the evaluation for an underlying genetic syndrome despite apparent absence of positive family history in these two patients.

Treatment response was different in the two patients with a complete response to medical therapy in Case 2 and poor response in Case 1. DA therapy is considered as first-line therapy in giant prolactinoma. According to review of published data from 97 patients, approximately 60% patients had shown complete hormonal response, while 74% had shown tumour shrinkage [1]. No pretreatment predictor of tumour response has been yet identified, but MEN-1 syndrome is reported to be associated with larger tumours (84% versus 24%) and treatment resistance (56% versus 10%) according to most of the published series [2, 8–10, 16, 17]. Prolactinomas in the recently published Dutch series responded well to DA treatment, but many of these were screening-detected microadenomas [8], suggesting a variability in response and a possible difference in responsiveness between small and large MEN-1 syndrome related lesions.

Unusual initial presentation in these patients prompted further evaluation and resulted in diagnosis of MEN-1 syndrome, despite an apparently negative family history. These two cases provide supportive evidence for the importance of genetic evaluation in young patients with giant prolactinomas. Our data also demonstrate the unpredictability of treatment response of these tumours. Vigilance and suspicion in index cases with MEN-1-like features can lead to early diagnosis and better care of these patients and their families.

## Figures and Tables

**Table 1:** Suggested biochemical and radiological screening in individuals with *MEN1* mutations [7].

Tumour	Age to begin(y)	Biochemical test annually	Imaging test (Time interval)

Parathyroid	8	Calcium, PTH	None

Pancreas	20	Gastrin	None
Gastrinoma			

Insulinoma	5	Fasting glucose, Insulin	None

Other pancreatic NET	<10	Chromogranin-A, PP, glucagon, VIP	MRI, CT or endoscopic US

Pituitary	5	Prolactin, IGF-1	MRI (every 3 y)

Adrenal	19	None unless >1cm lesion or symptoms	MRI or CT

Thymic and bronchial carcinoid	15	None	CT or MRI(1-2y)

## Data Availability

All patients' data are available and can be produced on request to access.

## Abbreviations

MEN: Multiple endocrine neoplasia
DA: Dopamine agonist
AIP: Aryl hydrocarbon receptor interacting protein
GH: Growth hormone
PRL: Prolactin
PNET: Pancreatic neuroendocrine tumour
HPT: Hyperparathyroidism.
